# Circulating concentrations of free triiodothyronine are associated with central adiposity and cardiometabolic risk factors in young euthyroid adults

**DOI:** 10.1007/s13105-022-00881-w

**Published:** 2022-04-06

**Authors:** Elisa Merchan-Ramirez, Guillermo Sanchez-Delgado, Cristina Arrizabalaga-Arriazu, Francisco M Acosta, Maria Jose Arias-Tellez, Manuel Muñoz-Torres, Jose V Garcia-Lario, Jose M Llamas-Elvira, Jonatan R Ruiz

**Affiliations:** 1 PROFITH “PROmoting FITness and Health Through Physical Activity” Research Group, Department of Physical and Sports Education, Sport and Health University Research Institute (iMUDS), Faculty of Sports Science, University of Granada, C/Menéndez Pelayo 32, 18016 Granada, Spain; 2 Pennington Biomedical Research Center, Baton Rouge, LA USA; 3 Department of Endocrinology, Fundación Hospital de Jove, Gijón, Spain; 4 Turku PET Centre, University of Turku, Turku, Finland; 5 Turku PET Centre, Turku University Hospital, Turku, Finland; 6 Department of Nutrition, Faculty of Medicine, University of Chile, Santiago, Chile; 7 Instituto de Investigación Biosanitaria (Ibs. Granada), Granada, Spain; 8 Department of Medicine, University of Granada, Granada, Spain; 9 CIBERFES, Instituto de Salud Carlos III, Granada, Spain; 10 Endocrinology and Nutrition Service, University Hospital San Cecilio, Granada, Spain; 11 UGC Laboratorio Clínico. University Hospital San Cecilio, Granada, Spain; 12 Nuclear Medicine Service, “Virgen de Las Nieves” University Hospital, Granada, Spain

**Keywords:** Thyroid function, Body composition, Cardiometabolic risk factors, Obesity, Euthyroid

## Abstract

Thyroid dysfunction is associated with classic cardiometabolic risk factors in humans. However, this relationship remains unclear in young euthyroid adults. The present work examines the associations of circulating thyroid hormones (THs) and thyroid-stimulating hormone (TSH) concentrations with body composition and cardiometabolic risk factors in young euthyroid adults. A total of 106 sedentary, euthyroid adults (72 women; 22 ± 2 years old) participated in this cross-sectional study. THs and TSH serum concentrations were determined in fasting conditions (6 h). Body composition (fat mass (FM), lean mass (LM), and visceral adipose tissue (VAT)) was determined by dual-energy X-ray absorptiometry, anthropometric parameters (weight, height, and waist circumference) were measured, and neck adipose tissue mass was quantified through computed tomography (CT) scanning. Cardiometabolic risk factors including fasting glucose and lipid metabolism markers, hepatic phosphatase and transaminases, and blood pressure were also assessed. Free triiodothyronine (FT3) concentration was positively associated with body mass index, LM, VAT, and waist circumference (all *P* ≤ 0.038). FT3 was also associated with glucose, insulin, HOMA-IR, fatty liver index, and blood pressure (all *P* < 0.024). All the associations were attenuated when adjusting for sex. In contrast, we found no associations of TSH or free thyroxine with any body composition parameter or cardiometabolic risk factors. In conclusion, FT3 is associated with central adiposity and cardiometabolic risk factors including insulin resistance, fatty liver index, and mean, systolic and diastolic blood pressure in young euthyroid adults. ClinicalTrials.gov identifier: NCT02365129.

**Supplementary Information:**

The online version contains supplementary material available at 10.1007/s13105-022-00881-w.

## Introduction

The prevalence of obesity has tripled since 1975 [1]. According to the data of the World Health Organization, in 2016, 39% of the adult population was overweight and 13% was obese [1]. Obesity is characterized by excessive accumulation of body fat and an altered energy metabolism. It is considered an important public health problem worldwide due to its association with increased risk of developing cardiovascular diseases, cancer, and many other pathologies [1].

Thyroid function plays an important role in body weight and energy metabolism regulation [2]. Pathological excess of thyroid hormones (THs), reflecting a hyperthyroidism state, causes weight loss, reduces cholesterol levels, and increases resting energy expenditure, lipolysis, and gluconeogenesis. Conversely, hypothyroidism is characterized by increased body weight and an unfavorable cardiometabolic profile [2]. Due to the important role of thyroid function in energy homeostasis [2] and cardiovascular health, several studies have investigated its relationship with obesity and cardiometabolic risk factors in euthyroid subjects [3, 4]. Both body mass index (BMI) and waist circumference (WC) are positively associated with serum-free triiodothyronine (FT3) and thyroid-stimulating hormone (TSH) levels in euthyroid subjects [5–7]. However, the relationship between obesity and free thyroxine (FT4) levels in euthyroid subjects is still controversial [5, 7]. On the other hand, previous studies have shown that moderately low (within the clinically considered normal range) FT4 and TSH levels are associated with increased glucose levels, insulin resistance, higher atherogenic lipid levels, and low-grade chronic inflammation in euthyroid subjects [8, 9]. However, the associations between insulin resistance and serum FT4 or TSH are still controversial [5, 10, 11]. Moreover, some studies have shown a positive association between serum FT3 levels and insulin resistance [5, 10, 11]. More recently, a thyroid hormone resistance index (the Parametric Thyroid Feedback Quantile based Index, PTFQI, based on the circulating concentrations of T4 and TSH) [12] has been shown to relate to the prevalence of obesity, metabolic syndrome, type 2 diabetes mellitus, and the incidence of diabetes-related deaths in the US general population, even in euthyroid subjects.

Despite the advances achieved in this field, the relationship of thyroid function with obesity and cardiometabolic risk factors in young euthyroid adults requires further investigation. BMI is the most used method to diagnose overweight and obesity. However, BMI-based diagnostic is not completely accurate due to the lack of fat mass (FM) and lean mass (LM) information. FM and LM have different physiological roles in metabolic health [13], and different FM/LM proportions at the same BMI are differentially related to insulin resistance and cardiometabolic risk factors [13]. Particularly, although the association between TH levels and body weight has been largely analyzed, the association between TH levels and body composition (i.e., FM and LM) is still unknown. Moreover, not only fat accumulation but also fat partitioning are an important risk factor for the development of cardiometabolic diseases. Visceral adipose tissue (VAT) causes insulin resistance and low-grade systemic inflammation and precedes pathological states such as metabolic syndrome and type 2 diabetes mellitus [14]. Crucially, despite that VAT mass is generally associated with total fat mass, it can largely vary between individuals with similar BMI and total fat mass [15]. Therefore, it is of great relevance to determine whether thyroid function is associated with VAT mass in euthyroid adults. Recently, other ectopic fat depots, such as neck adipose tissue (NAT) mass, have also been suggested to be a relevant indicator of cardiometabolic risk and pro-inflammatory profile [16, 17]. Since the thyroid gland function might be specially affected by this ectopic fat accumulation, to further understand the association between thyroid function and NAT mass is of clinical interest. Despite that thyroid function has been shown to be associated with classic cardiometabolic risk factors in euthyroid adults [10], whether it is also true in young euthyroid adults (18–25 years) remains to be determined. Furthermore, cardiorespiratory fitness is a better predictor of cardiovascular mortality than many classic cardiometabolic risk factors [18], and consequently, its relation with thyroid function might also bring light to the relation between the latter and cardiometabolic risk in young adults.

This study aimed to investigate the association of circulating thyroid hormones and TSH concentrations with body composition and cardiometabolic risk factors in young euthyroid adults.

## Materials and methods

### Participants

A total of 106 young adults (34 men, 72 women) aged 22 ± 2 years were included in this cross-sectional study (Table 1). All of them participated in the ACTIBATE study [19], an exercise-based randomized controlled trial (ClinicalTrials.gov ID: NCT02365129), from which baseline data were obtained to conduct this secondary analysis. All participants were young (18–25 years old) euthyroid adults (FT3, FT4, and TSH levels within the normal range: 2.5–4.94 pg/ml, 0.38–1.5 ng/dL, and 0.34–5.6 µUI/ml, respectively) who reported: (i) being sedentary (less than 20 min on less than 3 days per week of physical activity), (ii) having a stable body weight (changes < 3 kg in the last 3 months), (iii) not being smoker, (iv) not having acute or chronic diseases, and (v) not being pregnant. The study protocol and written informed consent were performed in accordance with the Declaration of Helsinki. The Ethics Committee of Human Research of the University of Granada (no 924) and the Servicio Andaluz de Salud (Centro de Granada, CEI-Granada, no 20.10.2014) approved the study. For logistic reasons, the measurements were conducted in eight evaluation waves, which means that participants were measured in different groups during the months of October, November, and December in 2015 and 2016.

**Table 1:** Characteristics of participants

	**All (*n* = 106)**		**Men (*n* = 34)**		**Women (*n* = 72)**		***P***
Age (years)	22.0	(2.1)	22.2	(2.1)	22.0	(2.1)	0.546
*Thyroid hormones*							
FT3 (pg/mL)	3.4	(0.4)	3.5	(0.3)	3.3	(0.4)	0.029
FT4 (ng/dL)	0.9	(0.1)	1.0	(0.1)	0.9	(0.1)	0.077
TSH (µUI/mL)	1.7	(0.8)	2.0	(0.9)	1.6	(0.6)	0.004
PTFQI	0.2	(0.3)	0.3	(0.2)	0.2	(0.2)	0.002
*Body composition and anthropometric measurements*							
Weight (kg)	70.6	(16.6)	83.7	(17.3)	64.7	(12.5)	< 0.001
Height (cm)	167.7	(8.9)	176.3	(6.9)	163.8	(6.7)	< 0.001
BMI (kg/m^2^)	24.9	(4.6)	26.9	(5.5)	24.0	(3.8)	0.002
Lean mass (kg)	41.4	(9.8)	52.8	(7.4)	36.3	(5.4)	< 0.001
Fat mass (kg)	25.2	(8.8)	25.5	(10.9)	24.9	(7.7)	0.511
Fat mass (%)	36.2	(7.2)	30.8	(7.2)	38.6	(5.7)	< 0.001
VAT mass (g)	340.6	(174.5)	432.4	(180.9)	299.6	(156.0)	< 0.001
WC (cm)	80.9	(13.8)	89.8	(15.1)	76.8	(11.1)	< 0.001
Total NAT mass (g)	4.0	(1.9)	4.3	(2.4)	3.8	(1.6)	0.231
*Cardiometabolic risk factors*							
Glucose (mg/dL)	87.5	(6.7)	89.6	(7.7)	86.5	(6.0)	0.027
Insulin (µUI/mL)	8.5	(5.0)	9.5	(6.7)	8.1	(4.0)	0.196
HOMA-IR	1.9	(1.3)	2.2	(1.7)	1.8	(1.0)	0.124
Total-cholesterol (mg/dL)	164.5	(29.4)	163.4	(32.4)	164.9	(28.2)	0.806
HDL-C (mg/dL)	52.3	(11.0)	45.3	(7.9)	55.5	(10.8)	< 0.001
LDL-C (mg/dL)	96.2	(24.6)	99.8	(27.1)	94.5	(23.4)	0.302
Triglycerides (mg/dL)	83.2	(47.5)	91.4	(48.3)	79.5	(47.0)	0.228
LDL-C/HDL-C ratio	1.9	(0.7)	2.3	(0.8)	1.8	(0.5)	< 0.001
TG/HDL-C ratio	1.7	(1.3)	2.2	(1.6)	1.5	(1.0)	0.003
TC/HDL-C ratio	3.2	(0.9)	3.7	(1.1)	3.0	(0.6)	< 0.001
APOA1 (mg/dL)	144.5	(27.8)	128.2	(18.1)	151.8	(28.4)	< 0.001
APOB (mg/dL)	70..4	(20.3)	73.9	(25.1)	68.8	(17.8)	0.239
C-reactive-protein (mg/L)	2.5	(3.3)	2.1	(2.3)	2.7	(3.6)	0.406
Homocysteine (µmol/L)	10.9	(3.4)	12.8	(3.9)	10.0	(2.7)	< 0.001
Leptin (µg/L)	6.2	(4.0)	4.7	(4.0)	6.9	(3.9)	0.006
Adiponectin (mg/L)	11.1	(8.0)	7.6	(5.7)	12.7	(8.4)	0.002
GGT (U/L)	18.8	(18.9)	28.9	(30.8)	14.1	(4.7)	< 0.001
ALP (U/L)	72.5	(18.9)	79.7	(19.5)	69.2	(17.9)	0.007
CMR score	0.0	(0.7)	-0.0	(0.7)	0.0	(0.6)	0.839
Fatty liver index	20.5	(24.7)	36.8	(31.7)	12.8	(15.5)	< 0.001
Systolic BP (mmHg)	116.2	(11.7)	124.7	(11.3)	112.4	(9.7)	< 0.001
Diastolic BP (mmHg)	71.0	(7.5)	72.8	(8.8)	70.1	(6.8)	0.094
Mean BP (mmHg)	85.9	(8.0)	89.9	(8.6)	84.1	(7.1)	< 0.001
VO_2_max (mL/kg/min)	40.9	(8.1)	44.8	(8.8)	39.1	(7.1)	0.001

Data are presented as mean and standard deviation*.* P value is from an independent samples *t*-test comparing men vs women. Abbreviations: *FT3,* free triiodothyronine;* FT4*, free Thyroxine; *TSH*, Thyroid-Stimulating Hormone; *PTFQI*, Parametric Thyroid Feedback Quantile based Index; *BMI*, body mass index; *LMI*, lean mass index, *FMI*, fat mass index; *VAT*, visceral adipose tissue; *NAT*, neck adipose tissue; *HOMA-IR*, homeostatic model assessment-insulin resistance; *HDL-C*, high-density lipoprotein cholesterol; *LDL-C*, low-density lipoprotein cholesterol; *TG*, triglycerides; *TC*, total cholesterol; *APOA1*, apolipoprotein A-1*; APOB*, apolipoprotein B; *GGT*, gamma-glutamyltransferase; *ALP*, Alkaline phosphatase; *CRM score*, cardiometabolic risk score; *BP*, blood pressure; *VO*_*2*_*max*, maximum oxygen consumption; *WC*, waist circumference

### Thyroid function

A blood draw was taken after a 6-h fast and after avoiding moderate (within 24 h) and vigorous (within 48 h) physical activity in the previous days. The blood draw took place between 8 am and 6:30 pm (no association was found between the time of the day when the samples were collected and the concentrations of FT3, FT4, and TSH, data not shown). Upon collection, blood was centrifuged and stored at 4 °C until analyses. FT3, FT4, and TSH circulating levels were determined using a Beckman Coulter DXI (33,880) chemiluminescent immunoassay system (Intra-assay CV FT3 = 5.7%, FT4 = 4.95%, TSH = 5.86%). In addition, we calculated the PTFQI — an indicator of resistance to thyroid hormones — which oscillates between − 1 and 1 [12]. Hence, negative values of this index indicate low TSH values by high inhibition of FT4, which indicates high sensitivity to FT4. In contrast, positive PTFQI values indicate high TSH by low inhibition of FT4, which means low sensitivity to FT4. Therefore, PTFQI evaluate the set point of the central regulation of THs concentration. PTFQI was calculated following Excel spreadsheet formula created by Laclaustra et al. [12].

### Body composition and anthropometric measurements

A whole-body dual-energy X-ray absorptiometry scan (Discovery Wi, Hologic, Inc., Bedford, MA, USA) was used to determine FM, LM, and VAT mass. Data were obtained from the Hologic APEX 4.0.2. (Hologic, Inc., Bedford, MA, USA) software. Weight and height of participants were measured without shoes and wearing light clothes using a SECA scale and stadiometer (model 799, Electronic Column Scale, Hamburg, Germany), and body mass index was calculated (kg/m^2^). Waist circumference (cm) was measured twice at the minimum perimeter, or at the medium point between the inferior rib and the iliac crest when the minimum perimeter was not apparent, with an inextensible metallic tape, in the standing position, and the average value was considered for further analyses.

NAT volume was determined by the analyses of the computerized tomography (CT) being part of a Positron Emission Tomography/Computerized tomography [Siemens 16 PET/CT scanner (Siemens, Erlangen, Germany)] that was carried out in the ACTIBATE study for quantifying brown adipose tissue volume [19]. The CT scans were performed from the atlas vertebra to the mid-chest region, and all of them were analyzed using the FIJI software (http://sourceforge.net/projects/bifijiplugins/) [20]. NAT volume was determined at the level of C5, as previously described [17]. We obtained the total NAT volume applying a radiodensity range of − 300 to − 10 Hounsfield units. NAT volume was then multiplied by a fat density coefficient of 0.9 g/ml to obtain the total NAT mass [21].

### Circulating cardiometabolic risk markers

Blood samples for determining cardiometabolic risk factors were obtained in the morning after an overnight fast (> 10 h), in resting conditions. Blood was later centrifuged, and serum aliquots were stored at − 80 °C until analyses. Fasting blood glucose concentration was determined by Beckman Coulter reagent OSR6521 in a Beckman Coulter AU5832 analyzer. Insulin concentration was determined by Chemiluminescent immunoassay of Beckman Coulter (33,410) with a DXI analyzer. The homeostatic model assessment index of insulin resistance (HOMA-IR) was calculated [22]. Concentrations of total cholesterol, triglyceride, Apolipoprotein A (APO A), and high-density lipoprotein cholesterol (HDL-C) were obtained with routine enzymatic methods with a Beckman Coulter AU5832 analyzer. To determine low-density lipoprotein cholesterol (LDL-C) concentration, the Friedewald formula was used [23]. Lipoprotein ratios (LDL-C/HDL-C, TG/HDL-C, and TC/HDL-C) were calculated as markers of dyslipidemia [24].

To measure the concentrations of C-reactive protein (CRP), Beckman Coulter reagent OSR6299 was used with an immunoturbidimetric method and then samples were processed in a Beckman Coulter AU5833 analyzer. Reagent from Axis-Shield Diagnostics Ltd (B08176) was used in a colorimetric method to determine homocysteine concentration in the Beckman Coulter AU5833 analyzer. The Beckman Coulter 447,730 reagent was used with an immunoturbidimetric method to determine the Apolipoprotein B (APO B) levels in the Beckman Coulter AU5833 analyzer. To measure gamma-glutamyl transferase (GGT) and Alkaline phosphatase (ALP) concentrations, Beckman Coulter reagent was used with a colorimetric method of the International Federation of Clinical Biochemistry (IFCC) in a Beckman Coulter AU5832 analyzer. Adiponectin concentration was determined with the MILLIPLEX MAP Human Adipokine Magnetic Bead Panel 2 (Catalogue # HADK2MAG-61 K; Intra-assay CV = 9%) and leptin concentration was determined with the MILLIPLEX MAP Human Adipokine Magnetic Bead Panel 1 (Catalogue # HADK1MAG-61 K; Intra-assay CV = 9%), both using Luminex MAP Technology platform (Luminex Corporation, Austin, TX, USA).

### Blood pressure

Blood pressure was assessed with an automatic sphygmomanometer Omron M2 (Omron Healthcare, Kyoto, Japan) on three different days, while the participant rested seated. The average of systolic and diastolic pressure on the three different days was calculated. Later, mean blood pressure (MBP) was calculated as follows [25]:

$$\mathrm{MBP}=\mathrm{Diastolic blood pressure}+0.33\times (\mathrm{Sistolic blood pressure}-\mathrm{Diastolic blood pressure})$$

### Combined cardiometabolic risk factors

#### Clustered score for cardiometabolic risk factors

A clustered score for cardiometabolic risk factors, based on diagnostic criteria of metabolic syndrome, was calculated [26]. Firstly, sex-specific Z-scores were obtained for waist circumference, mean blood pressure, HDL-C, triglycerides, and glucose. The cardiometabolic risk factors score was the average of the inverse Z-score of HDL-C and Z-scores of triglycerides, glucose, waist circumference, and mean blood pressure.

### Fatty liver index

The fatty liver index (FLI) was calculated with the following formula proposed by Bedogni et al. [27]:

$${FLI= (e}^{0.953*\mathrm{loge }\left(\mathrm{triglycerides}\right)+ 0.139*\mathrm{BMI }+ 0.718*\mathrm{loge }\left(\mathrm{GGT}\right)+ 0.053*\mathrm{waist circumference }- 15.745})/(1+{e}^{0.953*\mathrm{loge }\left(\mathrm{triglycerides}\right)+ 0.139*\mathrm{BMI }+ 0.718*\mathrm{loge }\left(\mathrm{GGT}\right)+ 0.053*\mathrm{waist circumference }- 15.745}) x 100$$

### Cardiorespiratory fitness assessment

Maximum oxygen consumption (VO_2_max) was determined by an incremental maximal graded treadmill (H/P/Cosmos Sport & Medical GMBH, Germany) walking test applying the modified Balke protocol [19, 28]. Participants did not consume stimulants 24 h before the test, fasted 3 to 5 h and did not perform vigorous or moderate physical activity (48 h and 24 h, respectively) before the test [19, 29]. The gas exchange was continuously registered by indirect calorimetry (Ultima CardiO2 cart, Medgraphics Corp, Minnesota, USA) with an oronasal mask (model 7400, Hans Rudolph Inc, Kansas City, MO, USA). VO_2_ was averaged every 5 s employing the Breeze Suite software (version 8.1.0.54 SP7, MGC Diagnostic®) and the highest observed VO_2_ after removing apparent artifacts were defined as the VO_2_max. Heart rate was continuously measured during the test with a heart rate monitor (Polar RS800X, Polar Electro Öy, Kempele, Finland).

### Statistical analyses

Data are reported as mean and standard deviation. To study the association of FT3, FT4, and TSH, and serum levels and PTFQI, with body composition parameters and cardiometabolic risk factors, we conducted simple linear regressions. All variables were transformed (square root transformation), except PTFQI, to normalize its distribution. Moreover, we also tested these associations using multiple linear regressions adjusting for sex. The sex × TSH/THs interaction effect on body composition/cardiometabolic risk factors was also analyzed for all the associations. Error alpha propagation by multiple comparisons correction (Benjamini–Hochberg procedure [30]) was performed. The Statistical Package for Social Sciences (SPSS, v. 22.0, IBM SPSS Statistics, IBM Corporation) was used to perform the analyses. The GraphPad Prism 8 (GraphPad Software, San Diego, CA, USA) was used to build the graphical plots. Statistical significance was set at *P* < 0.05.

## Results

**Fig. 1:**
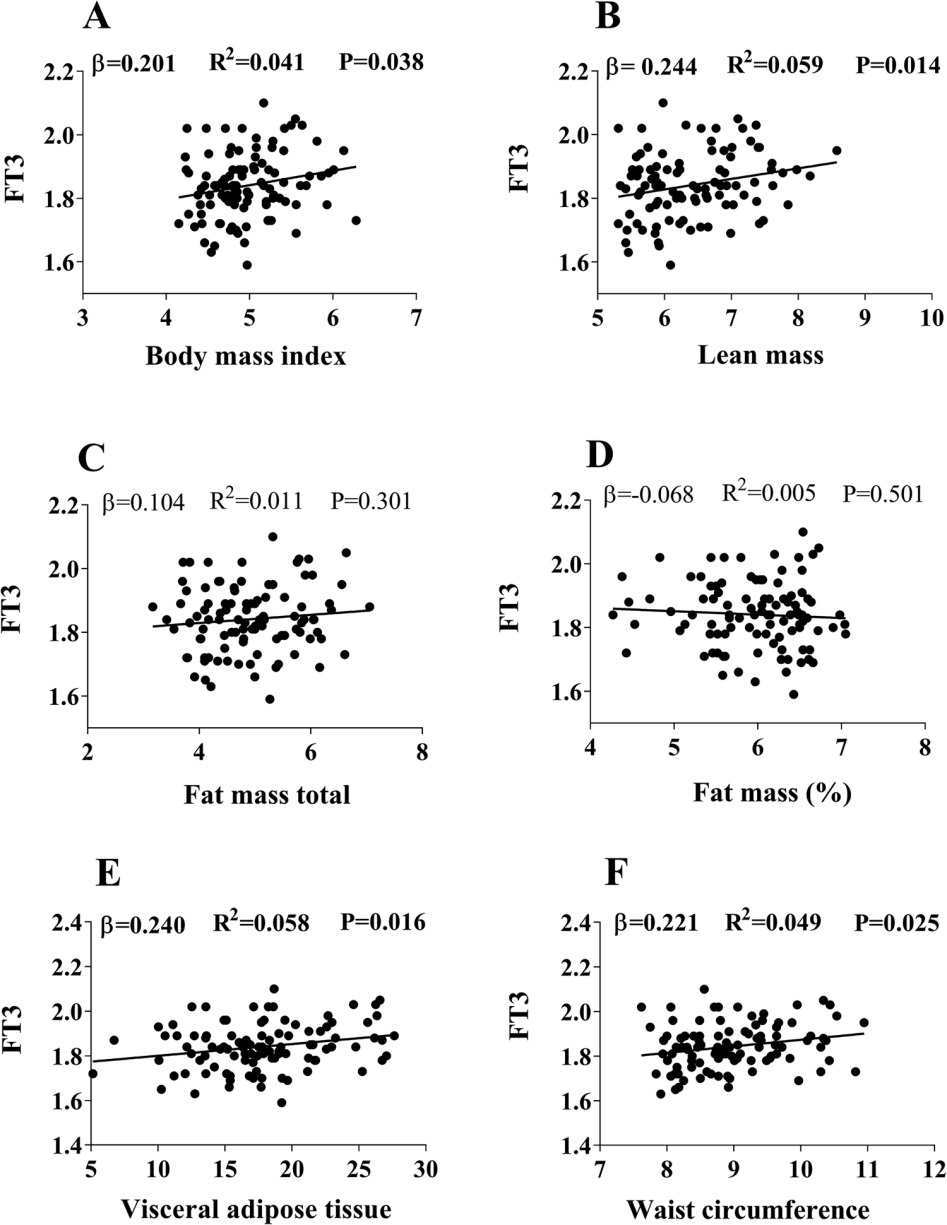
Association between serum levels of free triiodothyronine (FT3) and body composition parameters (Body mass index (**A**), lean mass (**B**), fat mass total (**C**), fat mass % (**D**), visceral adipose tissue (**E**) and waist circumference (**F**)). Standardized *β* coefficient, *R*^2^, and *P *value from linear regression analyses. All variables were square-root transformed (SQRT)

**Fig. 2:**
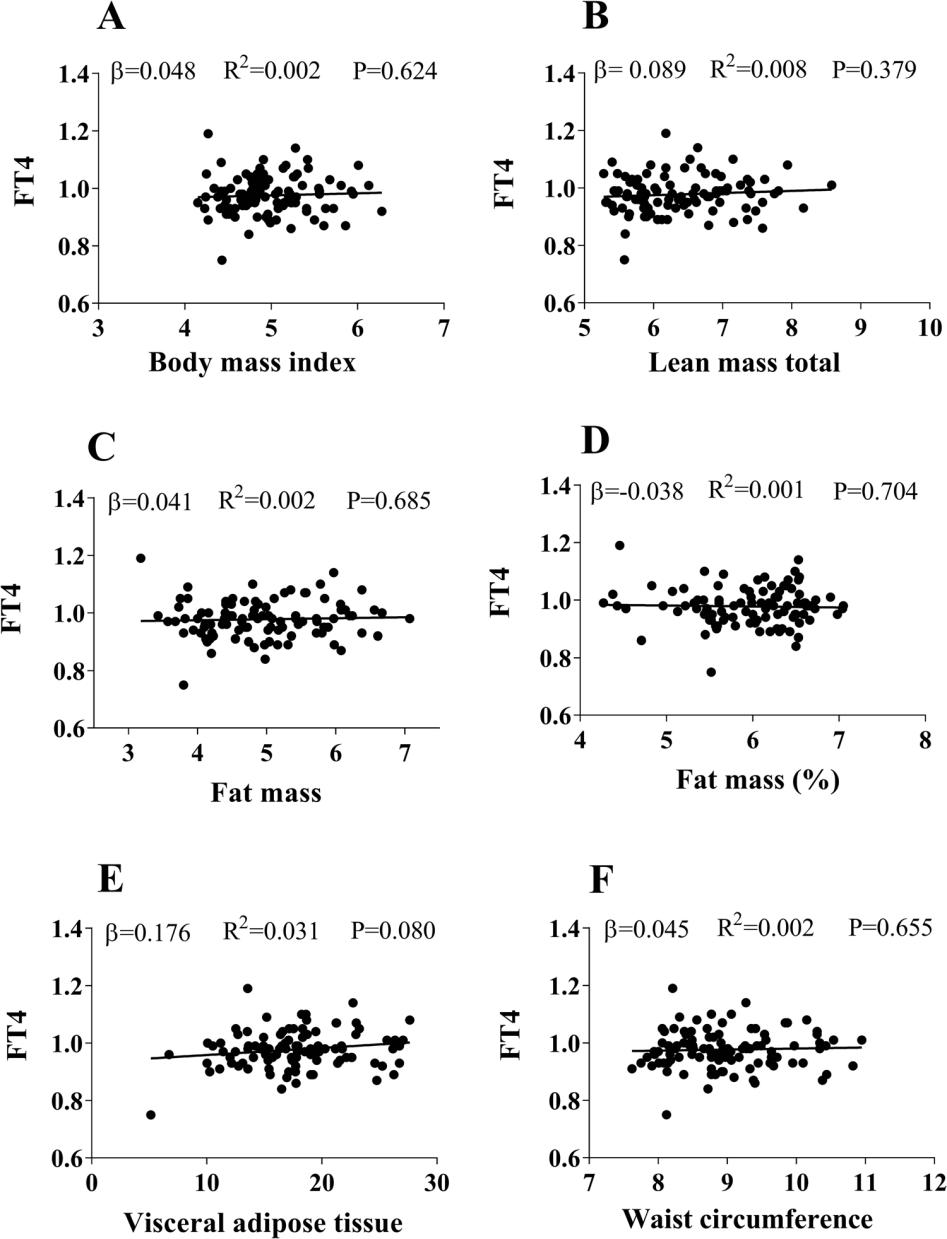
Association between serum levels of free thyroxine (FT4) and body composition parameters (Body mass index (**A**), lean mass (**B**), fat mass total (**C**), fat mass % (**D**), visceral adipose tissue (**E**) and waist circumference (**F**)). Standardized *β* coefficient, *R*^2^, and *P* value from linear regression analyses. All variables were transformed with square root transformation (SQRT)

**Fig. 3:**
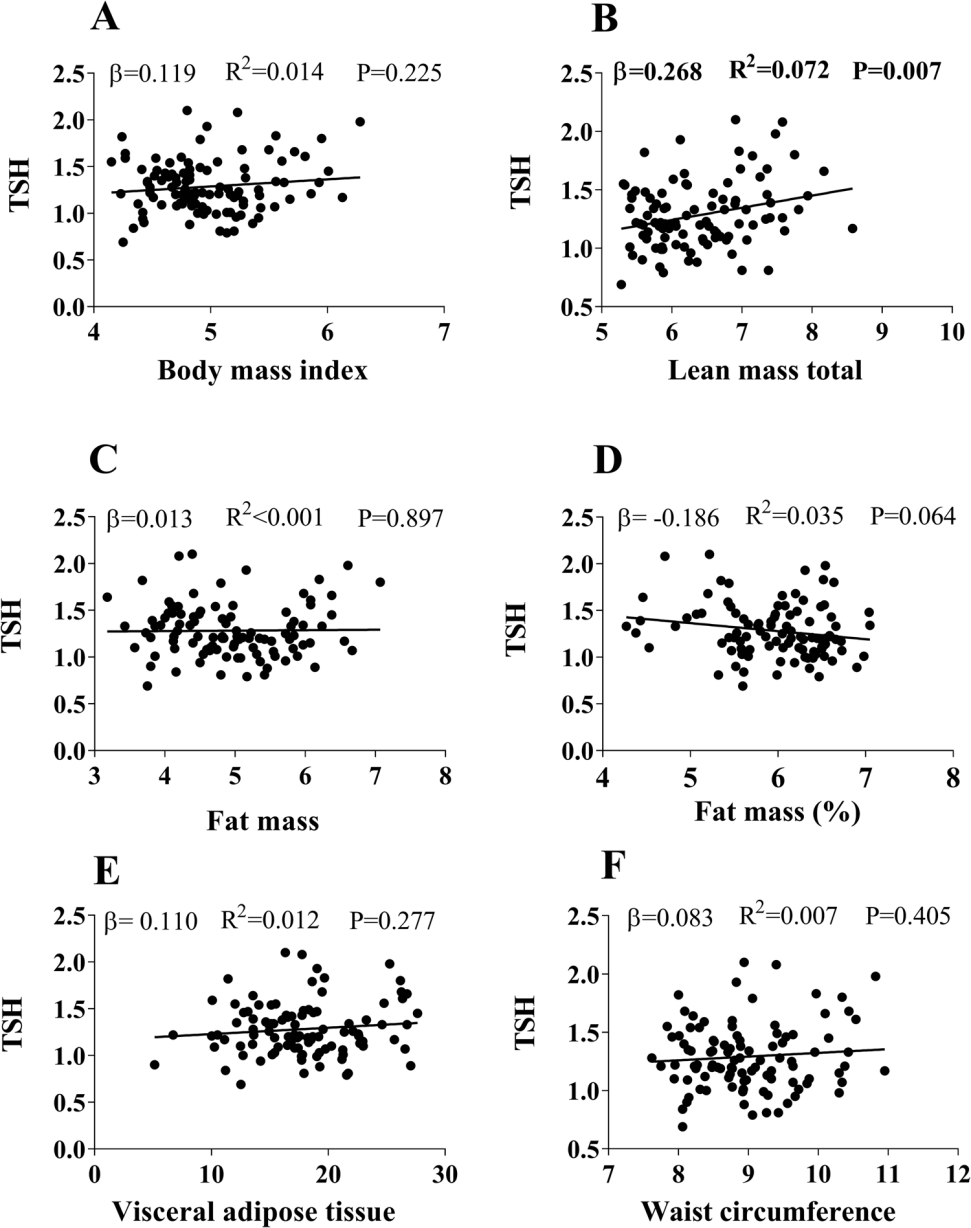
Association between serum levels of thyroid-stimulating hormone (TSH) and body composition parameters (Body mass index (**A**), lean mass (**B**), fat mass total (**C**), fat mass % (**D**), visceral adipose tissue (**E**) and waist circumference (**F**)). Standardized *β* coefficient, *R*^2^, and *P* value are from linear regression analyses. All variables were transformed with square root transformation (SQRT)

FT3 was positively associated with BMI (*β* = 0.201; *R*^*2*^ = 0.041; *P* = 0.038), LM (*β* = 0.244; *R*^*2*^ = 0.059; *P* = 0.014), VAT mass (*β* = 0.240; *R*^*2*^ = 0.058; *P* = 0.016), and WC (*β* = 0.221; *R*^*2*^ = 0.049; *P* = 0.025) (Fig. 1). Despite that there were no sex interaction effects, the associations between FT3 and VAT were attenuated after adjusting for sex (*β* = 0.174; *R*^*2*^ = 0.150; *P* = 0.072), and the association with BMI, WC, or LM disappeared (all *P* > 0.13) (data not shown). In contrast, FT4 was not associated with any body composition parameter (all *P* > 0.080) (Fig. 2). TSH was positively associated with LM (*β* = 0.268; *R*^*2*^ = 0.072; *P* = 0.007) (Fig. 3), but the association disappeared after adjusting for sex (*β* = 0.072; *R*^*2*^ = 0.619, *P* = 0.673, data not shown). PTFQI was also positively associated with LM (*β* = 0.243; *R*^2^ = 0.059; *P* = 0.015) and VAT mass (*β* = 0.210; *R*^*2*^ = 0.044; *P* = 0.036 (Figure S2), but these associations disappeared after having been adjusted for sex (*P* > 0.271, data not shown). No associations were found between FT3, FT4, TSH, or PTFQI and total NAT mass (Figure S3). The association analyses in men and women separately can be found in Table S1.

Figure S1 and Table 2 show the associations of THs and TSH with cardiometabolic risk factors. FT3 was positively associated with glucose, insulin, HOMA-IR, GGT, fatty liver index, systolic blood pressure, diastolic blood pressure, and mean blood pressure (all *P* < 0.024, Table 2). However, the associations between FT3 and cardiometabolic risk factors disappeared after adjusting for VAT mass (data not shown), except for the association between FT3 and systolic and mean blood pressure (*β* = 0.170; *R*^*2*^ = 0.373; *P* = 0.038 and *β* = 0.171; *R*^*2*^ = 0.347; *P* = 0.041, respectively). FT4 was negatively associated with adiponectin, and positively associated with systolic blood pressure, diastolic blood pressure, and mean blood pressure (all *P* < 0.047), but not with the rest of cardiometabolic risk factors (all *P* > 0.075; Table 2). A negative association was observed between TSH and HDL-C, and positive associations were found between TSH and LDL-C/HDL-C ratio, TG/HDL-C ratio, TC/HDL-C ratio, homocysteine, and SBP (all *P* < 0.039, Table 2), but no other association was observed between TSH and other cardiometabolic risk factors (all *P* > 0.051, Table 2). Cardiorespiratory fitness was not associated with any thyroid metabolism variables (*P* ≥ 0.662, data not shown). All the associations between FT4, TSH, and cardiometabolic risk factors remained after adjusting for VAT mass (data not shown). PTFQI was negatively associated with adiponectin, and was positively associated with homocysteine, systolic blood pressure, diastolic blood pressure, and mean blood pressure (all *P* < 0.023, Table 2). All the associations between THs and cardiometabolic risk factors were attenuated when adjusting for sex (Table S2).

**Table 2:** Association between serum levels of thyroid-stimulating hormone (TSH), thyroid hormones and Parametric Thyroid Feedback Quantile based Index (PTFQI) with cardiometabolic risk factors

	**FT3 (pg/mL)**			**FT4 (ng/dL)**			**TSH (µUI/mL)**			**PTFQI**		
	***β***	***R***^***2***^	***P***	***Β***	***R***^***2***^	***P***	***β***	***R***^***2***^	***P***	***β***	***R***^***2***^	***P***
**Glucose**	0.224	0.050	0.022	0.075	0.006	0.450	0.095	0.009	0.340	0.106	0.011	0.283
**Insulin**	0.263	0.069	0.007	0.141	0.020	0.153	0.082	0.007	0.409	0.120	0.014	0.225
**HOMA-IR**	0.269	0.072	0.006	0.134	0.018	0.174	0.089	0.008	0.371	0.122	0.015	0.218
**Total cholesterol**	-0.117	0.014	0.237	-0.019	< 0.001	0.848	0.060	0.004	0.543*	0.050	0.002	0.617
**LDL-C**	-0.073	0.005	0.461	-0.031	0.001	0.754	0.095	0.009	0.336	0.061	0.004	0.540
**HDL-C**	-0.192	0.037	0.051	-0.038	0.001	0.704	-0.225	0.051	0.021	-0.175	0.031	0.075
**Triglycerides**	0.051	0.003	0.610	-0.022	< 0.001	0.826	0.188	0.035	0.056	0.092	0.009	0.351
**LDL-C/HDL-C ratio**	0.072	0.005	0.468	0.004	< 0.001	0.970	0.206	0.043	0.035	0.152	0.023	0.124
**TG/HDL-C ratio**	0.131	0.017	0.185	0.006	< 0.001	0.951	0.236	0.056	0.016	0.145	0.021	0.141
**TC/HDL-C ratio**	0.095	0.009	0.336	0.026	0.001	0.793	0.239	0.057	0.015	0.191	0.036	0.052
**APOA1**	-0.124	0.015	0.211	-0.040	0.002	0.690	-0.193	0.037	0.051	-0.158	0.025	0.111
**APOB**	< 0.001	< 0.001	1.000	0.007	< 0.001	0.948	0.069	0.005	0.486*	0.063	0.004	0.528
**C Reactive protein**	0.109	0.012	0.270	0.121	0.015	0.219	-0.092	0.008	0.352	-0.018	< 0.001	0.854
**Homocysteine**	0.152	0.023	0.123*	0.024	0.001	0.810	0.250	0.062	0.011	0.222	0.049	0.023
**Leptin**	-0.008	< 0.001	0.937	-0.175	0.031	0.075*	-0.118	0.014	0.233	-0.189	0.036	0.054
**Adiponectin**	-0.129	0.017	0.193	-0.218	0.047	0.027	-0.168	0.028	0.089	-0.260	0.068	0.008
**GGT**	0.263	0.069	0.007	0.072	0.005	0.472	-0.025	0.001	0.805	0.044	0.002	0.657
**ALP**	0.149	0.022	0.131	0.064	0.004	0.518	-0.013	< 0.001	0.894	0.007	< 0.001	0.947
**CMR Score**	0.188	0.035	0.054	0.029	0.001	0.766	0.081	0.007	0.407	0.062	0.004	0.527
**Fatty liver index**	0.260	0.067	0.009	0.051	0.003	0.615	0.126	0.016	0.210	0.115	0.013	0.252
**Systolic BP**	0.306	0.094	0.001	0.194	0.038	0.047	0.202	0.041	0.039	0.283	0.080	0.003
**Diastolic BP**	0.246	0.061	0.011	0.238	0.057	0.014	0.061	0.004	0.534	0.237	0.056	0.015
**Mean BP**	0.301	0.091	0.002	0.243	0.059	0.012	0.136	0.018	0.168	0.285	0.081	0.003
**VO**_**2**_**max**	0.008	< 0.001	0.938	-0.044	0.002	0.662	0.030	0.001	0.766	0.019	< 0.001	0.848

Standardized *β* coefficient, *R*^*2*^, and *P* value from simple linear regression analyses. *Significant sex interaction. All variables were transformed (square root transformation), except PTFQI. Abbreviations: *FT3*, free triiodothyronine; *FT4*, free thyroxine; *TSH*, thyroid-stimulating hormone; *HOMA-IR*, homeostatic model assessment index of insulin resistance; *HDL-C*, high-density lipoprotein cholesterol; *LDL-C*, low-density lipoprotein cholesterol; *TG*, triglycerides; *APOA1*, apolipoprotein A-1; *APOB*, apolipoprotein B; *GGT*, gamma-glutamyltransferase; *ALP*, Alkaline phosphatase; *CRP*, C reactive protein; *CMR Score,* cardiometabolic risk score; *FLI,* fatty liver index; *BP*, blood pressure; *VO*_*2*_*max*, maximum oxygen consumption

The relationship of thyroid function with resting and peak heart rate was also explored and no associations were found (data not shown).

A significant *sex* × *FT3* interaction effect was found on homocysteine (*P* = 0.002) levels. The *sex* × *FT4* interaction was significant on leptin levels (*P* = 0.036). A significant *sex* × *TSH* interaction effect was also observed on total cholesterol (*P* = 0.017) and APOB (*P* = 0.014) levels. The association analyses in men and women separately can be found in Table S2.

None of the associations previously reported between thyroid function and body composition and cardiometabolic risk factors remained after alpha error propagation correction (data not shown).

## Discussion

The results of this study show that FT3 is positively associated with BMI, lean mass, central adiposity, and several cardiometabolic risks factors in young euthyroid adults. However, these associations were attenuated when adjusting for sex, and only some of them remained when analyzing men and women separately, which might point to a relevant role of sex in this relationship. On the other hand, we found no associations between FT4, TSH, and PTFQI and body composition parameters or cardiometabolic risk factors. These findings suggest that thyroid function, even when circulating levels of THs and TSH are within the normal range, might be related to body composition and cardiovascular risk in euthyroid young adults.

Several studies have analyzed the relationship between FT3 and adiposity [7, 11], but few have evaluated the connection between thyroid function and central adiposity in euthyroid young adults. Central adiposity is an important risk factor in the development of metabolic syndrome and cardiovascular disease [31]. We observed a positive association of FT3 with WC and VAT mass in young euthyroid adults. These results are in agreement with previous studies in euthyroid older obese subjects [8, 11]. Despite that VAT is commonly associated with ectopic fat deposition, we found no association of FT3, FT4, or TSH concentrations with total NAT mass. Moreover, we found no association of any body composition variable with FT4 or TSH, in agreement with the results obtained by Manji et al. [32]. Future studies should evaluate the relationship between thyroid function and central adiposity to understand the potential causes underlying the positive associations observed in studies [7].

Metabolic syndrome includes several risk factors for cardiovascular disease, namely, central obesity, elevated blood pressure, atherogenic dyslipidemia (high triglycerides and low HDL-C), and hyperglycemia. We observed the cardiometabolic risk score being positively associated with FT3 in euthyroid young adults. The relationship between thyroid function, insulin resistance, and metabolic syndrome is unknown as contradictory results have been published [33]. Indeed, De Pergola et al. [6], in line with our results, showed that FT3 was associated with WC, hyperinsulinemia, and other components of metabolic syndrome in overweight and obese euthyroid women. These findings suggest that thyroid function could predict the metabolic syndrome risk in euthyroid young adults. Furthermore, we observed that FT3 is associated with fasting glucose, insulin, and HOMA-IR. These findings have been widely reported in subjects with pathological circulating levels of THs [34, 35]. Ferranini et al. [36], showed that normal FT3 levels were associated with insulin resistance and glucose intolerance in subjects with BMI between 17 and 44 kg/m^2^.

The relationship between THs and blood pressure has also been extensively studied in pathological states, although this relationship still remains unclear in euthyroid subjects. In our study, THs and PTFQI were positively associated with systolic, diastolic, and mean blood pressure, and these results persist after adjusting for sex. However, no association was found between TSH and blood pressure, which concur with the finding by Roos et al. [37]. THs are important for vascular function. Both T3 and T4 act as a vasodilator on vascular smooth muscle cells [38]. Therefore, an increase of THs concentration in euthyroid subjects might represent a compensation for high blood pressure values. Alternatively, elevated concentrations of THs might itself contribute to increased blood pressure. Hyperthyroidism can produce tachycardia, increased heart contractility, elevated cardiac output, high systolic pressure, increased pulse pressure, and muscle weakness [39]. Therefore, elevated THs within the normal range could have the same effect as those in the hyperthyroid state, increasing blood pressure.

THs are important regulators of various metabolic processes, such as lipid metabolism [40]. The association between THs concentrations and serum lipids in pathological states has for long been studied [41, 42]. However, this relationship in euthyroid subjects is not clear yet. We observed a tendency to the negative association of FT3, TSH, and PTFQI with HDL-C, which is in agreement with Bakker et al. [43]. Hypothyroidism had been reported to be associated with an increased risk for dyslipidemia (especially higher serum levels of cholesterol, whereas their levels are reduced in hyperthyroidism) and atherosclerotic cardiovascular disease [44]. This phenomenon appears to be related to the cholesterol ester transfer protein (CETP) activity. CETP is positively regulated by THs, regulating the exchange of cholesteryl-ester between HDL-C and very-low-density lipoprotein (VLDL). Higher activity of CETP would imply an increased cardiovascular risk, related to elevation of VLDL and decreased HDL-C serum levels [40]. Thus, it is plausible that the tendency to an inverse association that we found of FT3, TSH, and PTFQI with HDL-C, is mediated by this mechanism, even in euthyroid subjects.

Cardiorespiratory fitness is currently recognized as an important marker of cardiovascular health, as it integrates the function of many physiological systems [18]. Therefore, we also investigated whether thyroid hormones are associated with cardiorespiratory fitness. Despite that FT3 was associated with many cardiometabolic risk factors, no association was found between TSH/THs and cardiorespiratory fitness in euthyroid adults.

### Limitations

The present results need to be interpreted with caution, since some limitations are present. First, it is a cross-sectional study and, therefore, no causality can be established. In addition, only healthy young adults are included in the study, so the results are not extrapolatable to older, younger, or unhealthy people. Furthermore, blood samples were measured at different times of the day, and due to the known circadian variations of THs levels, the results should be taken with caution. Moreover, despite that roughly two-thirds of the study sample were women, we did not standardize and neither controlled the menstrual cycle, which might have contributed to bias the results. Finally, despite that DXA is a valid instrument to assess body composition, it is limited to accurately assess VAT mass. Other more precise techniques such as magnetic resonance might have been more informative concerning the relationship between thyroid function and body composition. Likewise, other techniques such as lipoprotein profile analyses or hyperinsulinemic clamps for determining insulin resistance might be able to better detect relationships between thyroid function and cardiometabolic risk.

## Conclusion

In summary, FT3 seems to be associated with central adiposity and metabolic syndrome factors such as insulin resistance, mean, systolic and diastolic blood pressure, and fatty liver in euthyroid young adults. More studies are needed in this population to clarify the role of thyroid function in the development and prevention of cardiovascular diseases at early ages.

## Supplementary Information

Below is the link to the electronic supplementary material.

Supplementary file1 (PDF 950 KB)

## Data Availability

The datasets generated and/or analyzed during this study are available upon reasonable request.

## Abbreviations

ALP: Alkaline phosphatase
APOA1: Apolipoprotein A-1
APOB: Apolipoprotein B
BMI: Body mass index
BP: Blood pressure
CRM: Cardiometabolic
FM: Fat mass
GGT: Gamma-glutamyl transferase
HDL-C: High-density lipoprotein
HOMA-IR: Homeostatic model assessment index of insulin resistance
LDL-C: Low-density lipoprotein
LM: Lean mass
NAT: Neck adipose tissue
PTFQI: Parametric Thyroid Feedback Quantile-based Index
T3: Tri-iodothyronine
T4: Thyroxine
TC: Total cholesterol
TG: Triglycerides
THs: Thyroid hormones
TSH: Thyroid-stimulating hormone
VAT: Visceral adipose tissue
VO_2_max: Maximum oxygen consumption
WC: Waist circumference
