# Supplementary material for: Circulating concentrations of free triiodothyronine are associated with central adiposity and cardiometabolic risk factors in young euthyroid adults
Source: J Physiol Biochem. 2022 Apr 6;78(3):629–40. doi: 10.1007/s13105-022-00881-w (PMC9381449; doi:10.1007/s13105-022-00881-w)
Supplement: Supplementary file 1 — Supplementary file1 (PDF 950 KB) [file 13105_2022_881_MOESM1_ESM.pdf]

Circulating concentrations of free triiodothyronine are associated with central adiposity and cardiometabolic risk factors in young euthyroid adults

Journal of Physiology and Biochemistry

Elisa Merchan-Ramirez [1], Guillermo Sanchez-Delgado [1,2], Cristina Arrizabalaga-Arriazu [3], Francisco M. Acosta [1], Maria J. Arias-Tellez [1,4], Manuel Muñoz-Torres [5,6,7,8], Jose V. Garcia-Lario [9], Jose M. Llamas-Elvira [5,10], Jonatan R Ruiz [1].

[1]PROFITH "PROmoting FITness and Health through Physical Activity" Research Group, Department of Physical and Sports Education, Sport and Health University Research Institute (iMUDS), Faculty of Sports Science, University of Granada, Granada, Spain.

[2]Pennington Biomedical Research Center, Baton Rouge, LA, USA.

[3]Department of Endocrinology, Fundación Hospital de Jove, Gijón, Spain.

[4]Department of Nutrition, Faculty of Medicine, University of Chile, Santiago, Chile.

[5]Instituto de Investigación Biosanitaria (Ibs. Granada), Granada, Spain.

[6]Department of Medicine, University of Granada, Granada, Spain.

[7]CIBERFES, Instituto de Salud Carlos III, Granada, Spain.

[8]Endocrinology and Nutrition Service, University Hospital San Cecilio, Granada, Spain.

[9]UGC Laboratorio Clínico. University Hospital San Cecilio, Granada, Spain.

[10]Nuclear Medicine Service, "Virgen de las Nieves" University Hospital, Granada, Spain.

Corresponding authors: Elisa Merchan-Ramirez (E-mail: elisamerchan@ugr.es) and Jonatan R Ruiz (E-mail: ruizj@ugr.es), Instituto Mixto Deporte y Salud (iMUDS), University of Granada, C/Menéndez Pelayo 32, 18016 Granada, Spain.

Supplemental Material

Table S1. Association between serum levels of thyroid hormones and Parametric Thyroid Feedback Quantile based Index (PTFQI) with body composition by sex.

	**fT3 (pg/mL)**						**fT4 (ng/dL)**						**TSH (µUI/mL)**						**PTFQI**					
	**Men**			**Women**			**Men**			**Women**			**Men**			**Women**			**Men**			**Women**		
	**β**	**R^2**	**P**	**β**	**R^2**	**P**	**β**	**R^2**	**P**	**β**	**R^2**	**P**	**β**	**R^2**	**P**	**β**	**R^2**	**P**	**β**	**R^2**	**P**	**β**	**R^2**	**P**
**BMI**	0.059	0.003	0.741	0.201	0.040	0.091	-0.226	0.051	0.198	0.136	0.018	0.256	0.191	0.036	0.279	-0.070	0.005	0.559	0.033	0.001	0.852	-0.005	<0.001	0.968
**Lean mass**	0.101	0.010	0.581	0.141	0.020	0.251	-0.290	0.084	0.107	0.080	0.006	0.519	0.096	0.009	0.600	0.006	<0.001	0.961	-0.030	0.001	0.869	-0.001	<0.001	0.993
**Fat mass**	0.070	0.005	0.705	0.126	0.016	0.307	-0.211	0.045	0.246	0.211	0.044	0.084	0.095	0.009	0.603	-0.074	0.006	0.546	-0.019	<0.001	0.918	0.085	0.007	0.490
**Fat mass (%)**	0.058	0.003	0.753	0.061	0.004	0.619	-0.170	0.029	0.352	0.213	0.046	0.081	0.052	0.003	0.779	-0.092	0.008	0.457	-0.050	0.002	0.786	0.114	0.013	0.355
**VAT**	0.224	0.050	0.217	0.166	0.028	0.175	-0.157	0.025	0.391	0.271	0.073	**0.025**	0.107	0.011	0.560	-0.062	0.004	0.615	0.037	0.001	0.841	0.146	0.021	0.236
**WC**	0.041	0.046	0.818	0.214	0.047	0.078	-0.201	0.040	0.254	0.078	0.006	0.526	0.043	0.002	0.810	-0.089	0.008	0.465	-0.087	0.007	0.626	-0.048	0.002	0.698
**NAT mass**	0.152	0.023	0.449	0.137	0.019	0.300	0.062	0.004	0.757	0.055	0.003	0.678	-0.093	0.009	0.644	-0.043	0.002	0.747	0.020	<0.001	0.921	0.010	<0.001	0.937

Standardized β coefficient, R^2 and P value from linear regression analyses. All variables were transformed with square root transformation (SQRT), except PTFQI. Abbreviations: fT3, free Triiodothyronine; fT4, free Thyroxine; TSH, Thyroid-Stimulating Hormone; BMI, Body mass index; VAT, visceral adipose tissue; WC, waist circumference; NAT, Neck adipose tissue.

Table S2. Association between serum levels of thyroid hormones and Parametric Thyroid Feedback Quantile based Index (PTFQI) with cardiometabolic risk factors separating for sex.

	**fT3 (pg/mL)**						**fT4 (ng/dL)**						**TSH (µUI/mL)**						**PTFQI**					
	**Men**			**Women**			**Men**			**Women**			**Men**			**Women**			**Men**			**Women**		
	**β**	**R^2**	**P**	**β**	**R^2**	**P**	**β**	**R^2**	**P**	**β**	**R^2**	**P**	**β**	**R^2**	**P**	**β**	**R^2**	**P**	**β**	**R^2**	**P**	**β**	**R^2**	**P**
**Glucose**	0.014	<0.001	0.936	0.285	0.082	**0.017**	-0.132	0.017	0.458	0.146	0.021	0.229	0.059	0.004	0.739	0.026	0.001	0.832	-0.097	0.009	0.585	0.129	0.017	0.288
**Insulin**	0.289	0.084	0.097	0.234	0.055	0.052	0.190	0.036	0.281	0.086	0.007	0.481	0.207	0.043	0.240	-0.076	0.006	0.534	0.263	0.069	0.133	-0.014	<0.001	0.905
**Homa**	0.265	0.070	0.129	0.252	0.063	**0.036**	0.157	0.025	0.374	0.089	0.008	0.464	0.198	0.039	0.261	-0.071	0.005	0.562	0.235	0.055	0.181	-0.008	<0.001	0.950
**TC**	0.034	0.001	0.848	-0.195	0.038	0.105	-0.201	0.040	0.254	0.093	0.009	0.446	0.341	0.116	**0.049**	-0.139	0.019	0.252	0.141	0.020	0.426	0.012	<0.001	0.919
**LDL-C**	-0.017	<0.001	0.922	-0.140	0.019	0.249	-0.191	0.036	0.280	0.035	0.001	0.772	0.290	0.084	0.097	-0.093	0.009	0.443	0.094	0.009	0.596	-0.001	<0.001	0.993
**HDL-C**	-0.093	0.009	0.601	-0.121	0.015	0.317	-0.174	0.030	0.324	0.126	0.016	0.299	0.004	<0.001	0.982	-0.200	0.040	0.096	-0.096	0.009	0.588	-0.035	0.001	0.771
**Triglycerides**	0.205	0.042	0.244	-0.066	0.004	0.589	0.009	<0.001	0.958	-0.079	0.006	0.517	0.328	0.108	0.058	0.040	0.002	0.743	0.264	0.070	0.131	-0.057	0.003	0.642
**LDL-C/HDL-C ratio**	0.045	0.002	0.800	-0.027	0.001	0.823	-0.055	0.003	0.757	-0.062	0.004	0.611	0.216	0.047	0.219	0.054	0.003	0.658	0.129	0.017	0.469	0.009	<0.001	0.939
**TG/HDL-C ratio**	0.208	0.043	0.239	0.001	<0.001	0.993	0.067	0.005	0.706	-0.119	0.014	0.327	0.261	0.068	0.136	0.100	0.010	0.408	0.252	0.064	0.150	-0.051	0.003	0.673
**TC/HDL-C ratio**	0.098	0.010	0.508	-0.035	0.001	0.775	-0.023	0.001	0.896	-0.055	0.003	0.652	0.236	0.056	0.179	0.081	0.007	0.503	0.173	0.030	0.329	0.034	0.001	0.779
**APOA1**	0.025	0.001	0.890	-0.067	0.004	0.583	-0.264	0.070	0.137	0.096	0.009	0.429	-0.021	<0.001	0.907	-0.146	0.021	0.226	-0.130	0.017	0.471	-0.026	0.001	0.833
**APOB**	0.052	0.003	0.773	-0.067	0.005	0.580	-0.232	0.054	0.194	0.119	0.014	0.327	0.305	0.093	0.084	-0.177	0.032	0.142	0.106	0.011	0.556	-0.007	<0.001	0.955
**C Reactive protein (mg/L)**	0.000	<0.001	0.999	0.159	0.025	0.187	0.372	0.138	**0.030**	0.043	0.002	0.723	-0.030	0.001	0.865	-0.114	0.013	0.349	0.148	0.022	0.402	-0.061	0.004	0.618
**Homocysteine**	0.462	0.213	**0.006**	-0.117	0.014	0.335*	0.020	<0.001	0.912	-0.079	0.006	0.517	0.130	0.017	0.465	0.202	0.041	0.093	0.166	0.028	0.347	0.104	0.011	0.390
**Leptin**	0.034	0.001	0.851	0.081	0.007	0.507	-0.365	0.133	**0.034**	0.013	<0.001	0.913	0.034	0.001	0.850	-0.096	0.009	0.429	-0.205	0.042	0.244	-0.044	0.002	0.718
**Adiponectin**	0.075	0.006	0.674	-0.113	0.013	0.356	0.109	0.012	0.538	-0.296	0.087	**0.014**	-0.177	0.031	0.315	-0.030	0.001	0.808	-0.001	<0.001	0.997	-0.243	0.059	**0.044**
**GGT**	0.286	0.082	0.102	0.159	0.025	0.192	-0.063	0.004	0.724	0.072	0.005	0.558	-0.220	0.048	0.211	-0.134	0.018	0.273	-0.203	0.041	0.249	-0.042	0.002	0.733
**ALP**	0.051	0.003	0.774	0.125	0.016	0.303	0.088	0.008	0.620	-0.006	<0.001	0.961	-0.013	<0.001	0.940	-0.126	0.016	0.300	0.053	0.003	0.768	-0.128	0.016	0.290
**CMR Score**	0.200	0.040	0.257	0.196	0.038	0.099	-0.016	<0.001	0.929	0.058	0.003	0.629	0.133	0.018	0.454	0.057	0.003	0.632	0.107	0.012	0.545	0.051	0.003	0.671
**FLI**	0.141	0.020	0.426	0.234	0.055	0.056	-0.178	0.032	0.313	0.084	0.007	0.501	0.093	0.009	0.601	-0.075	0.006	0.544	-0.012	<0.001	0.948	-0.038	0.001	0.758
**Systolic BP**	0.263	0.069	0.139	0.226	0.051	0.057	-0.08	<0.001	0.922	0.191	0.036	0.109	0.085	0.007	0.637	0.084	0.007	0.484	0.135	0.018	0.453	0.168	0.028	0.157
**Diastolic BP**	0.374	0.140	**0.032**	0.150	0.022	0.210	0.152	0.023	0.397	0.254	0.064	**0.031**	0.017	<0.001	0.925	0.024	0.001	0.842	0.214	0.046	0.232	0.198	0.039	0.096
**Mean BP**	0.370	0.137	**0.034**	0.197	0.039	0.097	0.098	0.010	0.588	0.248	0.061	**0.036**	0.247	0.002	0.794	0.054	0.003	0.655	0.206	0.043	0.249	0.202	0.041	0.088
**VO2max**	0.075	0.006	0.678	-0.140	0.020	0.252	-0.040	0.002	0.824	-0.139	0.019	0.256	-0.074	0.005	0.683	-0.045	0.002	0.711	-0.071	0.005	0.695	-0.086	0.007	0.483

Standardized β coefficient, R^2 and P value from simple linear regression analyses. All variables were transformed with square root transformation (SQRT), except PTFQI. Abbreviations: FT3, free Triiodothyronine; FT4, free Thyroxine; TSH, Thyroid-Stimulating Hormone; HOMA-IR, Homeostatic model assessment index of insulin resistance; TC, Total cholesterol; HDL-C, High density lipoprotein cholesterol; LDL-C, low density lipoprotein cholesterol; TG, triglycerides; APOA1, Apolipoprotein A-1; APOB, Apolipoprotein B; GGT, gamma-glutamyltransferase; ALP, Alkaline phosphatase; CRP: C reactive protein; CMR Score, Cardiometabolic risk score; FLI, Fatty liver index; BP, blood pressure; VO_2max, maximum oxygen consumption.

Table S3. Association between serum levels of thyroid hormones and Parametric Thyroid Feedback Quantile based Index (PTFQI) with cardiometabolic risk factors adjusting for sex.

	TSH			FT4 (ng/dL)			FT3			PTFQI		
	**β**	**R^2**	**P**	**B**	**R^2**	**P**	**β**	**R^2**	**P**	**β**	**R^2**	**P**
**Glucose**	0.041	0.049	0.684	0.040	0.049	0.686	0.187	0.081	0.058	0.046	0.049	0.651
**Homa- IR**	0.059	0.020	0.567	0.116	0.030	0.247	0.252	0.078	**0.011**	0.091	0.025	0.378
**Insulin**	0.058	0.015	0.573	0.127	0.027	0.205	0.252	0.072	**0.012**	0.097	0.020	0.351
**Total Cholesterol**	0.070	0.005	0.496	-0.016	0.001	0.874	-0.118	0.014	0.246	0.061	0.004	0.559
**LDL-C**	0.075	0.0.015	0.467	-0.049	0.012	0.628	-0.098	0.019	0.333	0.035	0.011	0.739
**HDL-C**	-0.119	0.209	0.197	0.036	0.197	0.688	-0.105	0.206	0.252	-0.049	0.198	0.601
**Triglycerides**	0.158	0.047	0.119	-0.049	0.026	0.625	0.019	0.024	0.849	0.051	0.026	0.620
**APOA1**	-0.098	0.171	0.297	0.013	0.169	0.889	-0.042	0.170	0.654	-0.048	0.171	0.612
**APOB**	0.046	0.013	0.654	-0.007	0.011	0.943	-0.023	0.012	0.823	0.036	0.012	0.725
**C Reactive protein**	-0.087	0.009	0.396	0.132	0.019	0.190	0.123	0.016	0.226	-0.007	0.002	0.948
**Homocysteine**	0.164	0.166	0.085	-0.039	0.142	0.676	0.077	0.146	0.412	0.122	0.154	0.205
**Leptin**	-0.038	0.103	0.700	-0.124	0.117	0.196	0.061	0.105	0.526	-0.102	0.111	0.303
**Adiponectin**	-0.080	0.136	0.410	-0.162	0.155	0.085	-0.057	0.133	0.549	-0.167	0.155	0.087
**GGT**	-0.159	0.248	0.080	-0.007	0.224	0.934	0.172	0.252	0.055	-0.108	0.235	0.243
**ALP**	-0.083	0.068	0.407	0.024	0.062	0.808	0.102	0.071	0.301	-0.073	0.066	0.471
**CMR Score**	0.091	0.008	0.371	0.033	0.001	0.745	0.200	0.038	**0.046**	0.073	0.005	0.480
**Fatty liver index**	0.007	0.217	0.942	-0.025	0.218	0.784	0.171	0.245	0.059	-0.024	0.218	0.795
**Systolic BP**	0.076	0.244	0.397	0.108	0.250	0.218	0.211	0.281	**0.016**	0.145	0.258	0.110
**Diastolic BP**	0.021	0.024	0.833	0.217	0.069	**0.028**	0.223	0.071	**0.024**	0.210	0.064	**0.040**
**Mean BP**	0.050	0.113	0.608	0.188	0.145	**0.046**	0.240	0.166	**0.011**	0.201	0.147	**0.039**
**VO2max**	-0.056	0.095	0.573	-0.101	0.102	0.300	-0.071	0.096	0.471	-0.081	0.098	0.423

Linear regression analyses were performed. Standardized β coefficient, R^2 and P value are provided. All variables were transformed with square root transformation (SQRT), except PTFQI. Abbreviations: TSH, Thyroid-Stimulating Hormone; FT4, free Thyroxine; FT3, free Triiodothyronine; HOMA-IR, Homeostatic model assessment index of insulin resistance; HDL-C, High density lipoprotein cholesterol; LDL-C, low density lipoprotein cholesterol; TG, triglycerides; APOA1, Apolipoprotein A-1; APOB, Apolipoprotein B; GGT, gamma-glutamyltransferase; ALP, Alkaline phosphatase; CRP: C reactive protein; CMR Score, Cardiometabolic risk score; FLI, Fatty liver index; BP, blood pressure; VO_2max, maximum oxygen consumption.

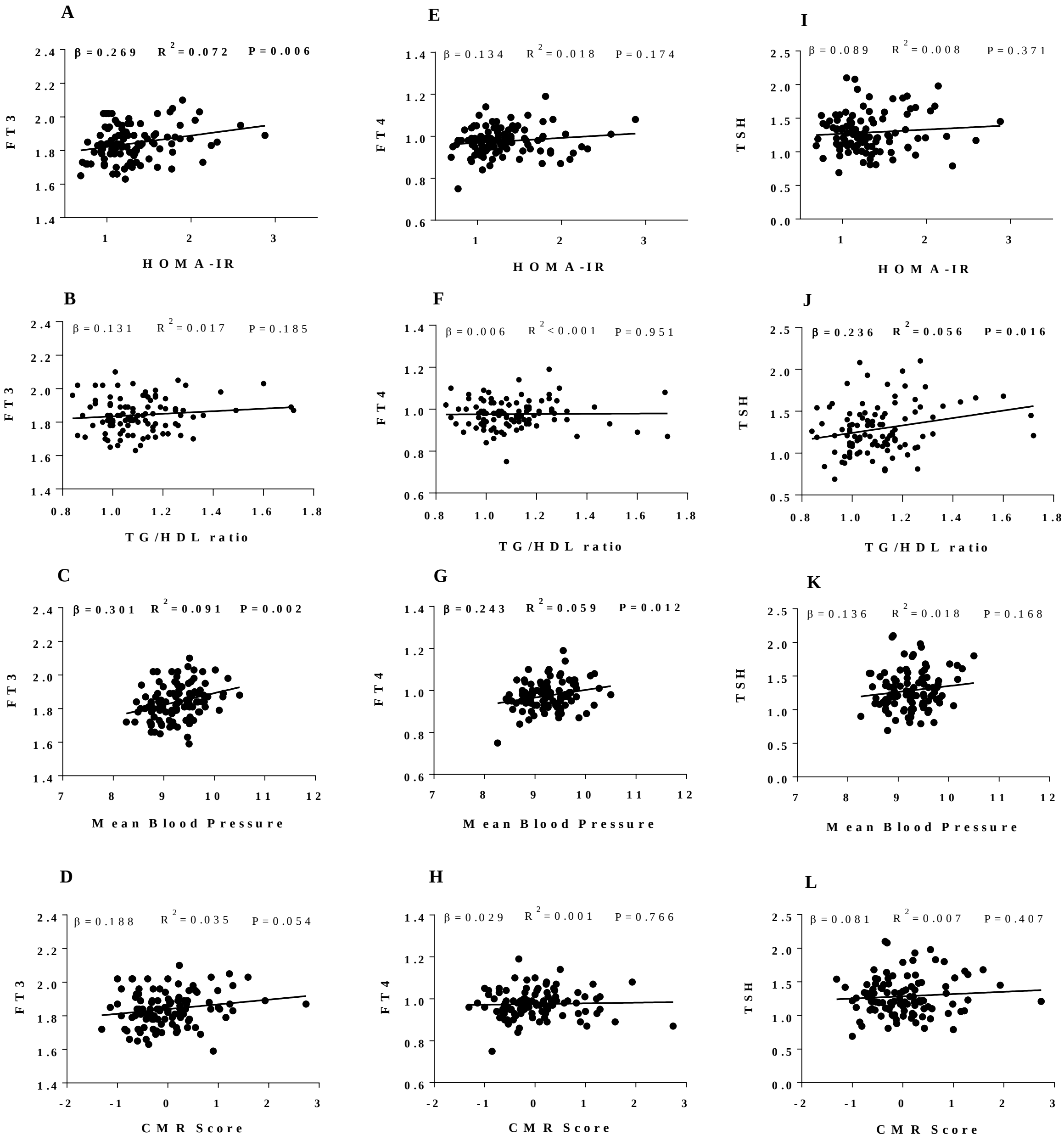

Figure S1. Association between serum levels of TSH and thyroid hormones with homeostatic model assessment of insulin resistance (HOMA-IR), Triglycerides (TG) / High-density lipoproteins cholesterol (HDL-C) ratio, mean blood pressure and cardiometabolic risk score (CMR Score). Standardized β coefficient, R^2 and P value from linear regression analyses. All variables were transformed with square root transformation (SQRT). Abbreviations: FT3, free Triiodothyronine; FT4, free thyroxine; TSH, Thyroid-Stimulating Hormone.

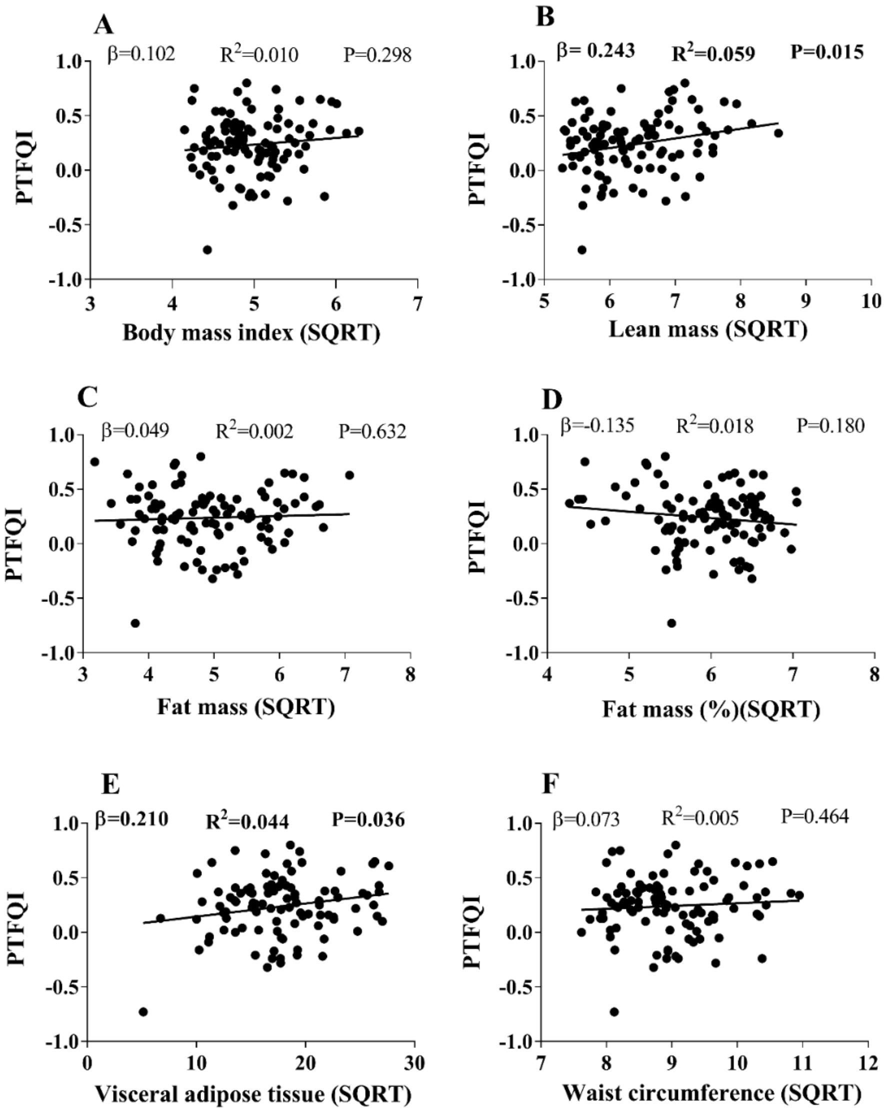

Figure S2. Association between Parametric Thyroid Feedback Quantile based Index (PTFQI) and body composition parameters. Standardized β coefficient, R^2 and P value from linear regression. All variables were transformed with square root transformation (SQRT), except PTFQI.

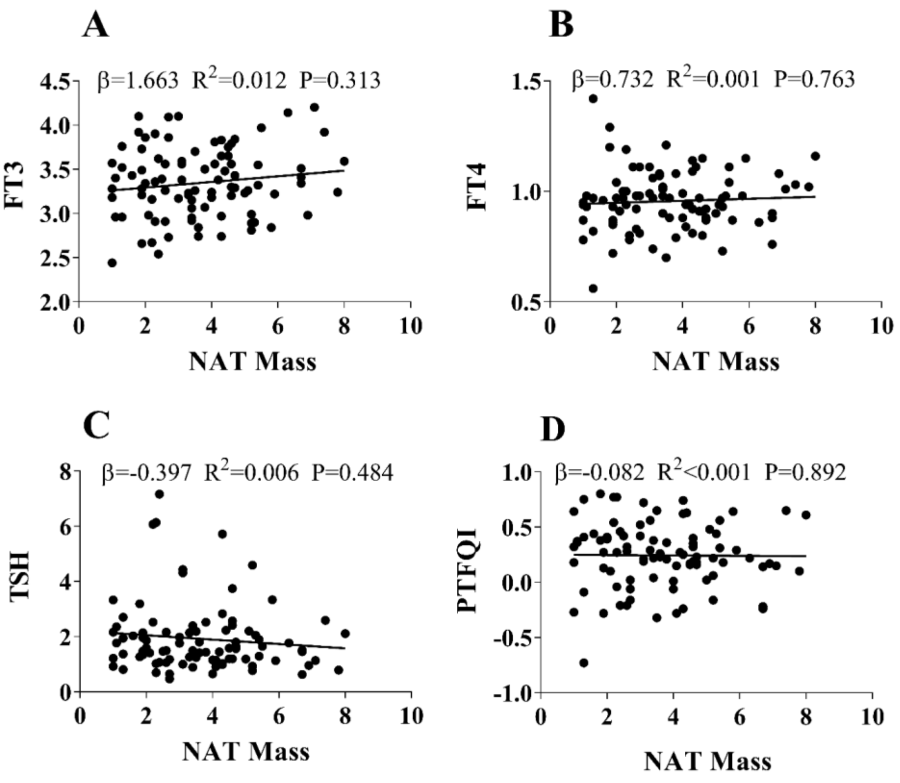

Figure S3. Association between serum levels of thyroid hormones and Parametric Thyroid Feedback Quantile based Index (PTFQI) with NAT Mass. Standardized β coefficient, R^2 and P value from linear regression. All variables were transformed with square root transformation (SQRT), except PTFQI. Abbreviations: TSH, Thyroid-Stimulating Hormone; fT4, free Thyroxine; fT3, free Triiodothyronine.
